# Revealing the composition of the eukaryotic microbiome of oyster spat by CRISPR-Cas Selective Amplicon Sequencing (CCSAS)

**DOI:** 10.1186/s40168-021-01180-0

**Published:** 2021-11-26

**Authors:** Kevin Xu Zhong, Anna Cho, Christoph M. Deeg, Amy M. Chan, Curtis A. Suttle

**Affiliations:** 1 Department of Earth, Ocean, and Atmospheric Sciences, The University of British Columbia, Vancouver, British Columbia Canada; 2 Department of Microbiology and Immunology, The University of British Columbia, Vancouver, British Columbia Canada; 3 Department of Botany, The University of British Columbia, Vancouver, British Columbia Canada; 4 Institute for the Oceans and Fisheries, The University of British Columbia, Vancouver, British Columbia Canada

**Keywords:** Eukaryotic microbiome, 18S rRNA gene, Microeukaryote, CRISPR-Cas, Taxon-specific single-guide RNA, gRNA target site, CasOligo, CCSAS

## Abstract

**Background:**

The microbiome affects the health of plants and animals, including humans, and has many biological, ecological, and evolutionary consequences. Microbiome studies typically rely on sequencing ribosomal 16S RNA gene fragments, which serve as taxonomic markers for prokaryotic communities; however, for eukaryotic microbes this approach is compromised, because 18S rRNA gene sequences from microbial eukaryotes are swamped by contaminating host rRNA gene sequences.

**Results:**

To overcome this problem, we developed CRISPR-Cas Selective Amplicon Sequencing (CCSAS), a high-resolution and efficient approach for characterizing eukaryotic microbiomes. CCSAS uses taxon-specific single-guide RNA (sgRNA) to direct Cas9 to cut 18S rRNA gene sequences of the host, while leaving protistan and fungal sequences intact. We validated the specificity of the sgRNA on ten model organisms and an artificially constructed (mock) community of nine protistan and fungal pathogens. The results showed that > 96.5% of host rRNA gene amplicons were cleaved, while 18S rRNA gene sequences from protists and fungi were unaffected. When used to assess the eukaryotic microbiome of oyster spat from a hatchery, CCSAS revealed a diverse community of eukaryotic microbes, typically with much less contamination from oyster 18S rRNA gene sequences than other methods using non-metazoan or blocking primers. However, each method revealed taxonomic groups that were not detected using the other methods, showing that a single approach is unlikely to uncover the entire eukaryotic microbiome in complex communities. To facilitate the application of CCSAS, we designed taxon-specific sgRNA for ~16,000 metazoan and plant taxa, making CCSAS widely available for characterizing eukaryotic microbiomes that have largely been neglected.

**Conclusion:**

CCSAS provides a high-through-put and cost-effective approach for resolving the eukaryotic microbiome of metazoa and plants with minimal contamination from host 18S rRNA gene sequences.

Video Abstract

**Supplementary Information:**

The online version contains supplementary material available at 10.1186/s40168-021-01180-0.

## Background

There is a growing interest in understanding how the composition of the microbiome affects the health of plants [1, 2] and animals [3–7], including humans [8, 9]. For example, in humans the gut microbiome is associated with both positive and adverse health effects, and changes in the microbiome have been linked to a number of diseases [10–13], such as obesity [14, 15], diabetes [16, 17], inflammatory bowel disease [18–21], cancer [22–25], cadiovascular disease [26, 27], and even mental illness [28–30]. As well, a wide span of biological, ecological, and evolutionary questions have been addressed through microbiome studies [3, 6–9, 31, 32]. Microbes have been shown to affect host metabolism [33], host immunity [34, 35], and human development [8, 36] including the brain [37, 38], and may even influence the evolution of animals and plants through microbe-host interactions [3, 7, 32, 39–45].

Microbiome studies have largely been facilitated through deep sequencing of ribosomal RNA gene fragments [46–49]; yet, our knowledge of the eukaryotic component of the microbiome, particularly protists, is relatively limited compared to that of prokaryotes [6, 49–53]. This is largely due to the challenge of profiling host-associated eukaryotic microbes, as the standard “universal” primers [54] used to amplify 18S rRNA gene sequences from eukaryotic microbes also amplify host 18S rRNA gene sequences, which will dominate the sequencing library [46, 52, 55].

A number of approaches have been used to minimize contamination by host 18S rRNA gene sequences. For example, primers can be designed that will not amplify host 18S rRNA sequences, but will amplify sequences from microeukaryotes (e.g., reference 56–58); alternatively, other marker genes can be targeted such as the ITS region of fungi [59]. However, designing primers to amplify ribosomal RNA gene sequences from a broad range of microeukaryotes, but not the host, can be challenging.

Another approach is to use primers to block amplification of host 18S rRNA sequences to study the eukaryotic microbiome [60]. Such “blocking primers” typically use a short blocking-oligonucleotide with a modified 3′ end that binds to the 18S rRNA gene of the host, and prevents “universal” 18S primers from amplifying host sequences [60]. Such an approach has been successfully applied to krill [60], fish [61, 62], coral [63], primates [64], shrimp [65, 66], flying squid [67], mosquitos [68, 69] and Pacific oysters [57], although a large proportion of the sequences can still be host-derived (e.g., up to 92% in coral, 42% in krill, and 45% in fish) [57, 63, 71]. This approach also requires designing and optimizing the blocking primers for each animal host, which remains a challenge [70, 71].

Recently, a method involving the usage of non-metazoan (UNonMet) primers [58] was developed [70, 71] and was shown to be effective in coral and humans [70, 72]. This “non-metazoan primers” method employs a nested-PCR approach which involves a two-step PCR procedure. The first-PCR step uses UNonMet primers [58] to generate ~600-bp fragments of 18S rRNA gene that are specific to microeukaryotes but not to metazoans; the products from the first PCR are reamplified using the “universal” 18S primers to produce a shorter 18S rRNA gene fragment [70]. This method has the advantage of not requiring host-specific primer design, but based on in silico analysis cannot be used for sponges and ctenophores [70].

Here, we describe CRISPR-Cas Selective Amplicon Sequencing (CCSAS), an alternative approach to resolve the eukaryotic microbiome of metazoa and plants. Clustered regularly interspaced short palindromic repeats (CRISPR) and the CRISPR-associated protein 9 (Cas9) system provides bacteria and archaea adaptive immunity against viruses and plasmids by cleaving invading double-stranded (ds) DNA [73]. The sequence-specific cleavage is performed by Cas9 endonuclease in the presence of guide RNA (gRNA). This gRNA is a duplex comprising a trans-activating RNA (tracrRNA) that is a scaffold for binding the Cas9 protein, and an approximately 20 nucleotide (nt) crispr RNA (crRNA) guide sequence that is complementary to the DNA target site [74–77]. Cas9 can be programmed to target any DNA sequence by modifying the 20-nt guide sequence [77, 78]. Due to its precision in DNA cutting, the simplicity in programming and the ability to artificially fuse the gRNA duplex (tracrRNA-crRNA) into a single-guide RNA (sgRNA) [77], CRISPR-Cas9 has emerged as a powerful tool in a wide variety of applications [78, 79]. CCSAS leverages this tool by using a custom sgRNA to direct Cas9 to specifically cut host 18S rRNA gene sequences in the region flanked by “universal” primers. The cleaved host 18S fragments contain only a 3′ or 5′ primer-binding region, resulting in short single-stranded (ss) DNA products produced by PCR, which are removed during the preparation of the sequencing library. This results in a library highly enriched in 18S amplicons from microeukaryotes, allowing for high-resolution surveys of the taxonomic composition of eukaryotic microbes associated with any eukaryotic host.

## Results

### Design of the taxon-specific sgRNA

The key to CCSAS is the 20-nt guide sequence of gRNA that directs Cas9 to selectively cut the 18S rRNA gene sequences of the host, but not those of the associated microeukaryotes. We developed CasOligo (https://github.com/kevinzhongxu/CasOligo), an R package that contains the algorithm Cas9.gRNA.oligo1(), which identifies 20-nt sequences in the 18S rRNA gene region spanned by “universal” primers that can serve as target-sites for gRNA, and which are complementary to the sgRNA’s guide sequence. The selected gRNA and sgRNA duplex thus dictates the specificity of the sgRNA-CRISPR-Cas complex, allowing a user to easily synthesize a taxon-specific sgRNA.

**Fig. 1:**
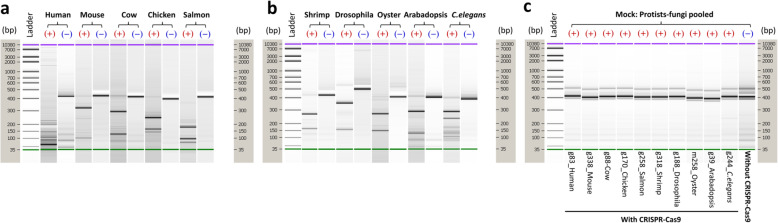
Agilent Bioanalyzer gel images of 18S amplicons from ten model organisms (**a** and **b**) and an artificial community of protists and fungi (**c**) to which Cas9 with the taxon-specific sgRNA (as shown in Table S2) was either added (**+**) or not (**−**). Gel bands show the amplicon length in base pairs (bp) relative to a DNA ladder. The labels on the *X*-axes of panel **c** indicate the ID of the taxon-specific sgRNAs and its corresponding host to target

To validate the target-specificity of sgRNA, taxon-specific sgRNAs were designed and tested for 18S rRNA sequences from each of the following ten model organisms: human (*Homo sapiens*), salmon (*Salmo salar*), shrimp (*Solenocera crassicornis*), chicken (*Gallus gallus domesticus*), cow (*Bos taurus*), mouse (*Mus musculus*), fruit fly (*Drosophila melanogaster*), rock cress (*Arabidopsis thaliana*), oyster (*Crassostrea gigas*), and the nematode (*Caenorhabditis elegans*), as well as being tested against an artificially constructed (mock) community composed of nine protists and fungi (Table S1 and S2). The results showed that the CRISPR-Cas9 treatment effectively cleaved the host 18S amplicons, while amplicons from the mock community of protists and fungi remained intact (Fig. 1). Comparisons using qPCR with and without CRISPR-Cas9 treatment showed that only 0.6% to 3.5% of the intact 18S amplicons remained after CRISPR-Cas9 cutting (Fig. S1). Thus, the sgRNAs effectively targeted host sequences, while leaving sequences from microeukaryotes intact.

### Using CCSAS to reveal host-associated microeukaryotic populations

**Fig. 2:**
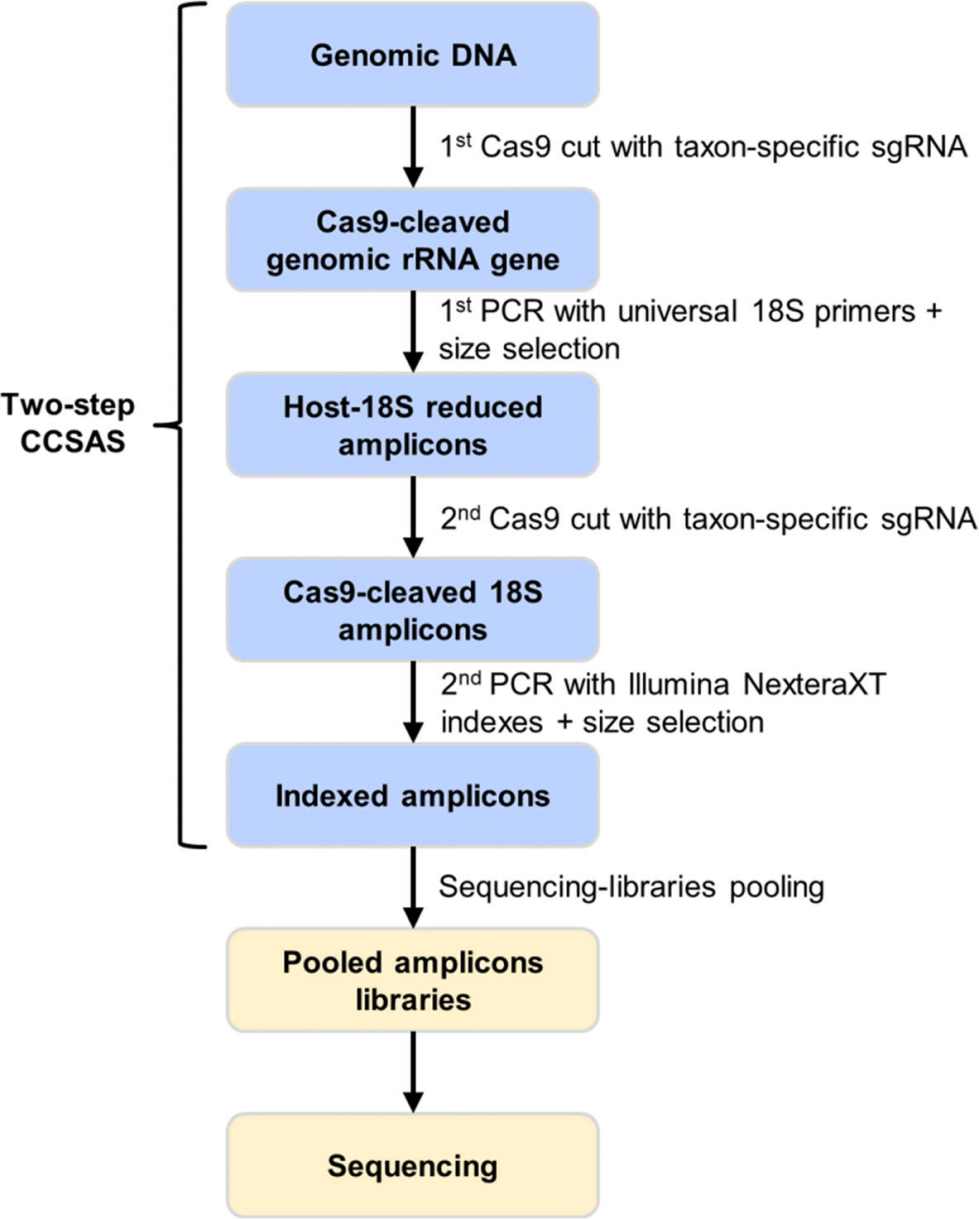
Workflow for two-step CRISPR-Cas Selective Amplicon Sequencing (CCSAS) to study the composition of the host-associated eukaryotic microbiome

The next step was to evaluate the effectiveness of Cas9 when complexed with the host-specific sgRNA. After CRISPR-Cas9 treatment, about 0.6% to 3.5% of the remaining 18S amplicons were still host-derived, but in most cases still dominated the sequencing library (data not shown). Hence, to further reduce the host-derived 18S rRNA gene sequences, we introduced a two-step CRISPR-Cas9 procedure (Fig. 2). First, Cas9 with a taxon-specific sgRNA that is complementary to the host 18S rRNA gene sequence at the 20-nt target-site is used to cut the host genomic 18S rRNA gene and then the remaining uncut 18S sequences are amplified using PCR. Any amplification of the cut fragments yields short pieces of ssDNA that are removed during size-selection clean-up step using SPRI magnetic beads. Second, following the first size selection, another Cas9 cut, PCR amplification, and size selection are conducted (Fig. 2), resulting in almost the complete removal of host 18S amplicons, while leaving the protistan and fungal amplicons intact. This allows for high-resolution characterization of the composition of the microeukaryotic community with a fraction of the sequencing effort typically used.

**Fig. 3:**
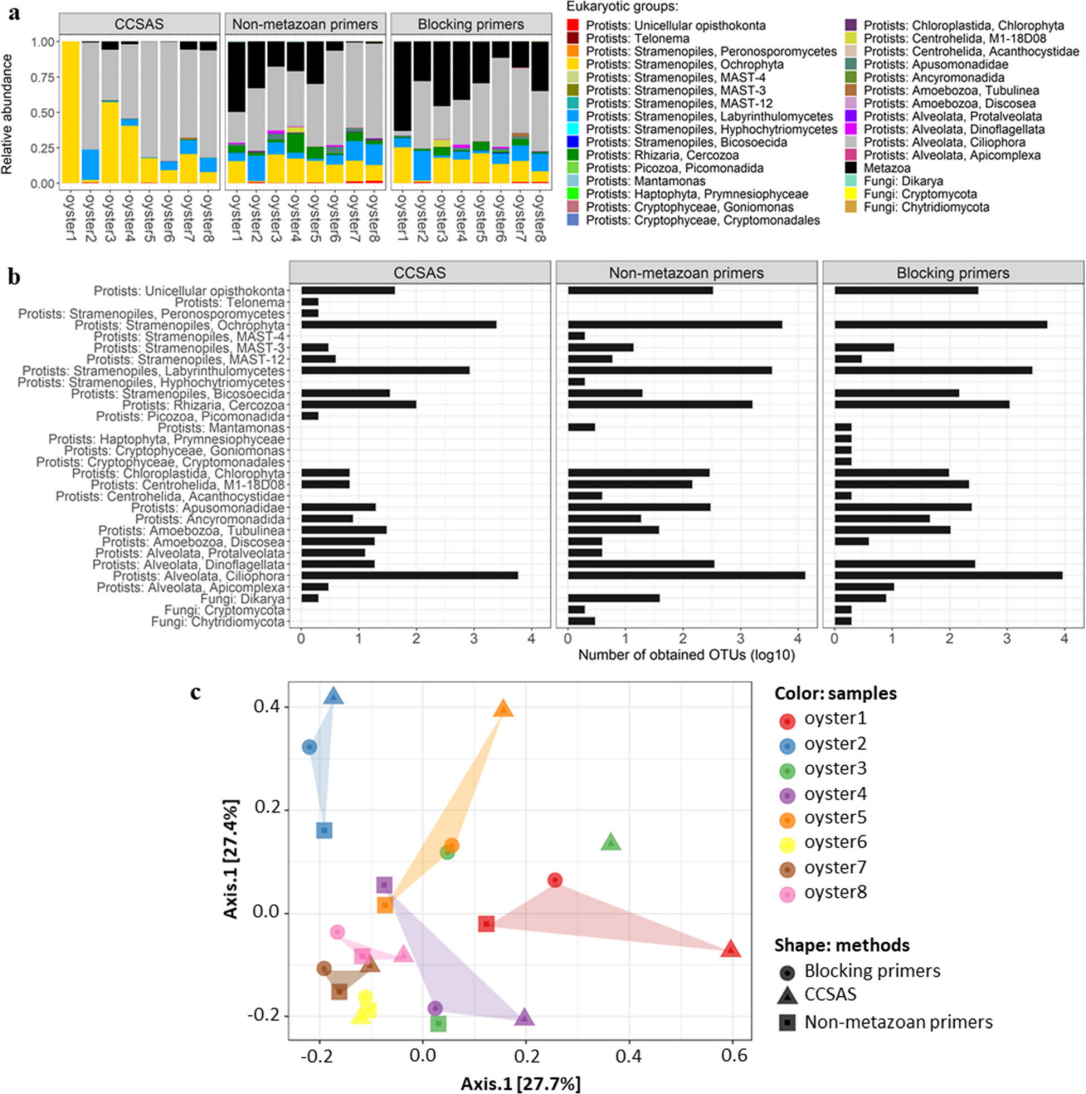
Panel **a** shows the relative abundances of eukaryotic groups in eight oyster samples revealed using deep-sequencing of 18S amplicons of rRNA genes. The methods are based on using non-metazoan primers (NM), blocking primers (BP), and CCSAS that combines 18S “universal” primers and cleavage using CRISPR-Cas9. Panel **b** shows the comparison of the number of 18S rRNA gene OTUs summed for the eight oyster samples for each microeukaryotic group. Panel **c** shows the Principal Coordinate Analyses (PCoA) of the community structure of the microeukaryotic 18S rRNA gene sequences in oyster spat. The PCoA ordinates the weighted Unifrac matrix that are based on the presence/absence and the relative abundance of microeukaryotic OTUs. Symbol colors represent the oyster samples, and the shapes indicate different methods (CCSAS, NM, BP)

We applied two-step CCSAS to examine the eukaryotic microbiome from eight different samples of oyster spat (*C. gigas*) collected from a hatchery that was experiencing mortality events. The results showed that using CCSAS in conjunction with “universal” 18S primers resulted in almost the complete removal of oyster 18S amplicons, while leaving the protistan and fungal amplicons intact and highly enriched for sequencing (Fig. S2; Fig. 3a). With CCSAS, the percentage of sequences from metazoa (mostly assigned to oysters, although some were from nematodes in the order Monhysterida; Fig. S2) was at most 7.4%, while in three out of eight samples, sequences from metazoa were undetectable (Fig. 3a). In contrast, with non-metazoan and blocking primers, up to 48.5% and 62.9% of sequences, respectively, were still from metazoa (Fig. 3a), primarily oysters (Fig. S2). When compared to non-metazoan and blocking primers, CCSAS revealed all the major eukaryotic microbial groups including members of the Ochrophyta, Labyrinthulomycetes, and Ciliophora (Fig. 3a-b). Nevertheless, given that there are differences in primer design among the three methods, as well as differences in PCR conditions (Table S3) [54, 57, 63, 70, 102], it is not surprising that there were differences among the taxa detected (Fig. 3b). For example, CCSAS detected the genus *Telonema*, peronosporomycetes in the Stramenopiles, and Picomonadida in the Picozoa, while non-metazoan and blocking primers did not. Yet, CCSAS did not detect the MAST4-group of stramenophiles or hyphochytriomyctes, while non-metazoan primers did, and prymnesiophytes, cryptophytes, and fungi in the phylum Cryptomycota and the division Chytridiomycota were only revealed by the blocking primers. As well, members of the genus *Mantamonas* and the family Acanthocystidae were detected by the non-metazoan and blocking primers, but not by CCSAS (Fig. 3b). Additionally, using CCSAS, the relative abundances of cercozoans and dinoflagellates were less than with the other methods (Fig. 3a). Thus, amplification with “universal” 18S primers combined with CCSAS had less contamination by host sequences and revealed some additional taxa compared to non-metazoan and blocking primers; however, there were also some taxa that were absent using CCSAS. Nonetheless, the composition of the eukaryotic microbiome detected by the three methods was quite similar (ANOSIM: *r*^2^ = 0.131, *p* value = 0.032, *n* = 8; PERMANOVA: *r*^2^ = 0.145, *p* value = 0.175, *n* = 8), and taxa that were not detected by one or more methods were always a minor component of the overall community.

**Fig. 4:**
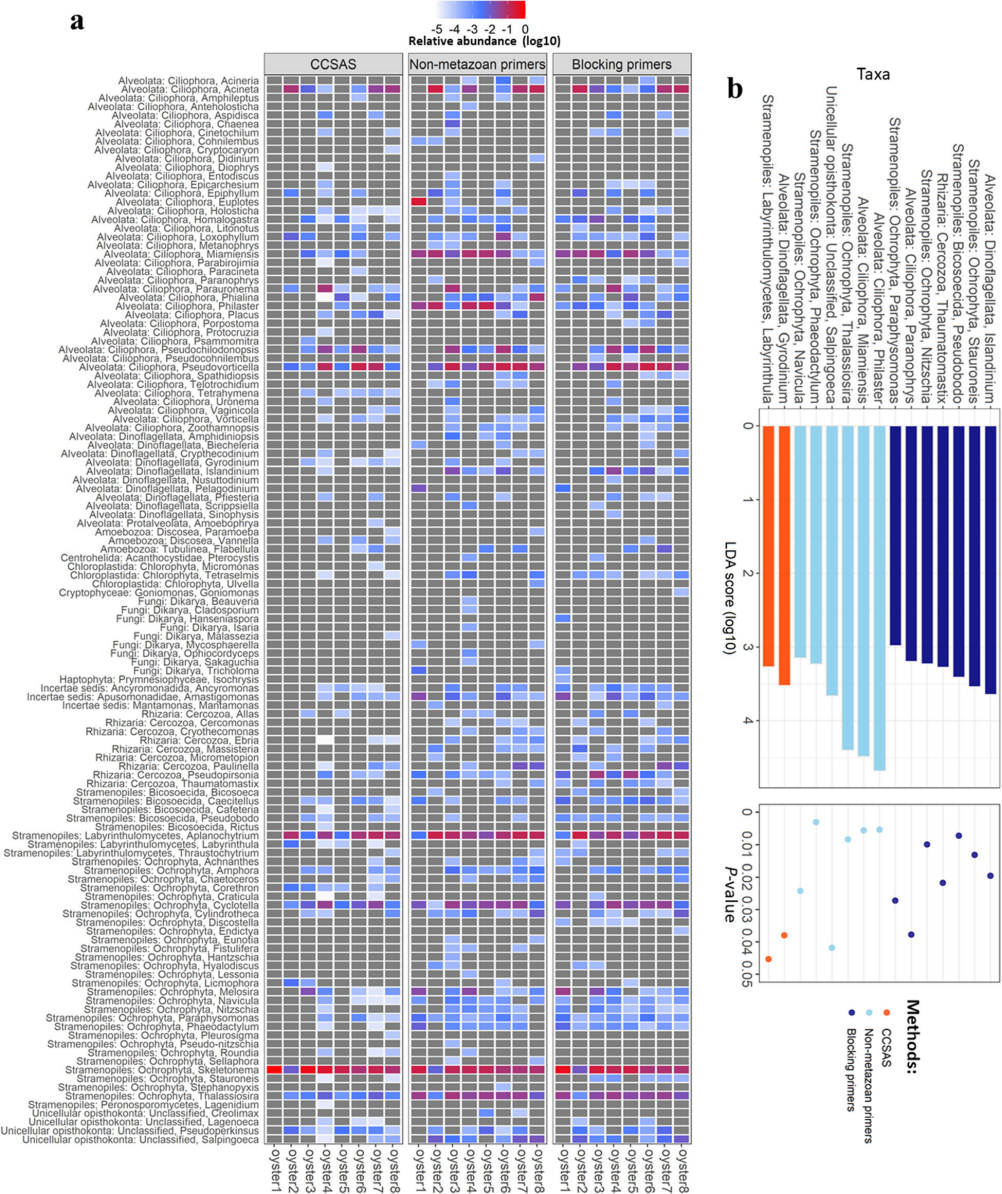
Heatmap (**a**) showing the relative abundance of 18S rRNA genes in microeukaryotic genera in oyster spat using the three methods. In panel **b**, linear discriminant analysis effect size (LEfSe) reveals differentially detected microeukaryotic genera in oysters among the three methods. The histogram shows LDA scores calculated for differences in genus-level abundances among microeukaryotic 18S rRNA genes for the three methods, and the dot plot shows the *p* values that determines the LDA significance

Moreover, the community structure was quite similar among the three methods for four of eight oyster samples (i.e., Oyster 2, 6, 7, and 8; Fig. 3c), although the genus-level relative abundances were variable across microeukaryotic genera between oysters and methods (Fig. 4a). Differences in genera detected among methods (Fig. 4b) included a dinoflagellate (*Gyrodinium*) and labyrinthulomycete (*Labyrinthula*) that occurred in relatively higher abundance using CCSAS. In contrast, compared to CCSAS, the blocking primers resulted in relatively higher abundance of several genera including a dinoflagellate (*Islandinium*), ochrophytes (*Stauroneis*, *Nitzschia*, and *Paraphysomonase*), a ciliophoran (*Paranophrys*), a cercozoan (*Thaumatomasix*), and a bicosoecid (*Pseudobodo*). Finally, compared to CCSAS, using the non-metazoan primers resulted in higher relative abundances of other genera belonging to ciliophora (*Philaster* and *Miamiensis*), a unicellular opisthokont (*Salpingoeca*), and ochrophytes (*Navicula*, *Phaeodactylum*, and *Thalassiosira*). Nonetheless, despite the differences among methods, the composition of the microeukaryotic communities was quite similar among methods.

### Database of gRNA target sites for metazoa and plants

**Fig. 5:**
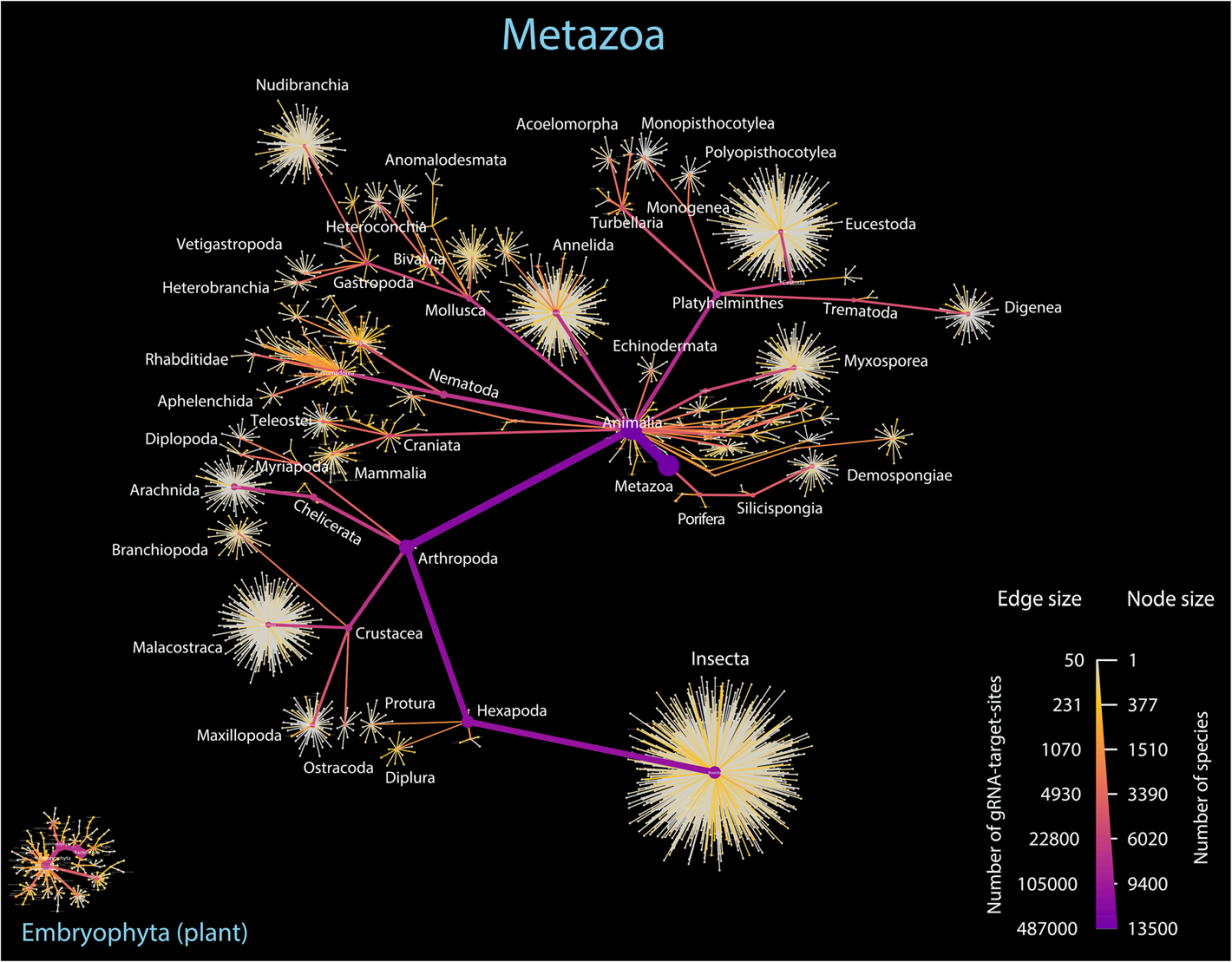
Illustration showing the number and taxonomic distribution of gRNA target sites for metazoa and plants that are available for 18S rRNA sequences in the SILVA SSU database v119 [80]. These gRNA-target-site oligonucleotide sequences are used for designing and synthesizing the taxon-specific and the CRISPR-Cas9-compatible sgRNAs that are used to guide CRISPR-Cas9 to cut the 18S rRNA genes of metazoan or plant hosts, but not those of microeukaryotes (protists and fungi). The node size indicates the number of species at each corresponding taxonomic level, while the size of the edge presents the number of gRNA target sites. Nodes and edges with the highest values are purple, while the smallest ones are gray. Only taxa with more than 50 gRNA target sites per taxon are shown

To enable CCSAS to be easily applied for characterizing eukaryotic microbiomes in a wide range of metazoa and plants, we used CasOligo to identify gRNA target sites for 99.6% of the 15907 metazoa and plant taxa (metaphyta of Embryophyta group) in the SILVA SSU database [80] (*version 119, released on 24 July 2014*) (Fig. 5). For each taxon, we identified between 3 and 217 (average 33) gRNA target sites that are compatible with the CRISPR-Cas9 system (Fig. S3); of these, between 1 and 214 targeted the putative host 18S sequence, but not protistan or fungal sequences. Thus, the database provides a wide selection of gRNA-target-site sequences from which to design taxon-specific sgRNAs.

Although it is not possible to design a “universal” sgRNA that targets all metazoa and plants, but not microeukaryotes, some sgRNAs target broad taxonomic groups (Fig. S4). For example, based on in silico analysis, sgRNA_058534 targets 3099 species from 22 classes and families of Animalia, primarily 72.7% of the 4014 Insecta species in SILVA (Fig. S4). CasOligo can also be used to retrieve gRNA-target-site sequences for specific taxa by entering the species name of the host using the function, search.db.byname(). Nonetheless, it is best to identify the taxon-specific gRNA target site based on the 18S rRNA gene sequence of the host, because the action of CRISPR-Cas9 is sequence-specific and the gRNA-target-site database does not cover all sequence variants for a specific taxon.

## Discussion

CCSAS provides a new way to obtain high-resolution taxonomic data for the eukaryotic microbiomes of plants, animals, and other metazoa. By employing CRISPR-Cas9 with taxon-specific gRNAs, the background of host 18S sequences is greatly reduced or eliminated; thus, CCSAS requires much less sequencing than other methods to obtain high-resolution taxonomic data for the eukaryotic microbiome. Moreover, the creation of a database of gRNA target sites, and the primary gRNA-target-site oligonucleotide design functions of the CasOligo package, makes it easy to profile the eukaryotic microbiome of metazoa and plants. We identified taxon-specific gRNA target sites for 99.6% of the taxa in the SILVA database, with an average of 33 taxon-specific gRNA target sites per taxon, showing that CCSAS can be applied to nearly all metazoa and plants. Additionally, the CasOligo package provides an oligonucleotide design function, Cas9.oligo.search2(), that can be used to design custom sgRNAs for any gene for which the sequence is known, and for which there is a reference database for comparison, so that the specificity of the sgRNA can be ascertained. This includes genes encoding other regions of rRNA, such as the 16S and 23S rRNA genes, or metabolic genes (e.g., COX1). Thus, CCSAS makes it possible to study the genetic diversity of any gene in complex systems, including genes that are rare, by removing any sequence that would otherwise dominate the data. The sequence-specific removal of any amplicon has a wide range of applications, including pathogen diagnosis, and studies of symbiosis and microbiome therapy.

There are a few considerations in applying CCSAS to microbiome studies. First, gRNA can recognize the wrong target [81–83], which might lead Cas9 to cut some protistan and fungal sequences, or incompletely cleave host sequences. This problem can be minimized by careful design of the gRNA, and in silico analysis against the most comprehensive databases of 18S gene sequences. Second, efficient sequencing requires effective removal of the cut host amplicons. This can be accomplished by optimizing the size selection of SPRI magnetic beads or may reduce sequencing efficiency, or adapting other methods for size selecting DNA fragments. Third, there are inherent amplification biases associated with PCR [84]; thus, the accuracy of differences in the relative abundances of specific sequences using CCSAS, or any other PCR-based approach is unknown. Fourth, the design of host-specific gRNA target sites is only as good as the available 18S rRNA gene references for microeukaryotes. However, the design of gRNA target sites will continue to improve as SSU sequence databases continue to expand. Despite these caveats, CCSAS can be used to obtain high-resolution data on the composition of eukaryotic microbiomes with relatively low-sequencing effort. Moreover, it has broad application because gRNA target sites can be identified for thousands of host species.

Our study revealed that most micro-eukaryotes in farmed Pacific oyster spat from British Columbia, Canada, were diatoms in the genera *Skeletonema*, *Thalassiosira*, and *Cyclotella*, ciliates in the subclass Scuticociliatia, and labyrinthulomycetes in the genus *Aplanochytrium* (Fig. 3a; Fig. 4a). In another study of the eukaryotic microbiome in farmed adult Pacific oysters in France [57], diatoms, dinoflagellates, and ciliophorans in the genus *Trichodina* (subclass Peritrichia) were abundant. As oysters filter-feed on protists and bacteria [85], it is not surprising that diatoms and dinoflagellates are enriched in the oyster microeukaryotic microbiome. Notably, in oyster spat in BC, ciliates from the subclass Scuticociliatia accounted for up to ~78.2% of the 18S rRNA using CCSAS, and up to ~40% using the other two methods (Fig. S5). The scuticociliates were from diverse genera including *Cohnilembus*, *Entodiscus*, *Homalogastra*, *Metanophrys*, *Miamiensis*, *Paranophrys*, *Parauronema*, *Philaster*, *Porpostoma*, *Pseudocohnilembus*, and *Uronema* (Fig. S5; Fig. 4a). Members from some of these genera can be opportunistic pathogens that cause disease (e.g., Scuticociliatosis, Brown-band disease) in a broad range of marine animals such as crustaceans, mollusks, corals, and fish including seahorses and sharks [86–95]. Scuticociliates from the genera *Paranophrys* and *Uronema* have been reported to cause disease in oysters in the USA [96] and Australia [97, 98], respectively. Members of the genera *Paranophrys* and *Uronema* were not detected or were in low abundance in all eight oyster samples from BC (Fig. S5; Fig. 4a). Another putative pathogen, the ciliate *Miamiensis avidus*, was in relatively high abundance across all oyster samples (Fig. S5).

Although there were differences in the taxonomic profiles of microeukaryotes generated using the three methods, it is important to stress that the relative abundances of most taxa were quite similar using the different approaches. The most striking difference among methods was the much lower relative abundance of sequences from metazoa that was detected using CCSAS, indicating less contamination from host-derived sequences. As well, each of the methods resolved some taxa of microeukaryotes that the other methods did not; however, the taxa that were selectively detected were present in relatively low abundance. These differences are not surprising given that each primer set differs in its mismatches among taxa [99, 100]. Consequently, relative abundance data must be interpreted cautiously. Nonetheless, our data show the utility of using CCSAS to investigate the eukaryotic microbiome of animals in a complex environment, and that oyster spat harbor potential pathogens including the scuticociliates *Miamiensis avidus*, *Paranophrys* sp., and *Uronema* sp.

## Conclusions

CCSAS is a powerful tool with which to investigate the composition of the eukaryotic microbiome for a vast array of host organisms. Relative to approaches using non-metazoan or blocking primers, CCSAS provides similar resolution of the eukaryotic microbial community, but with much less contamination by sequences from host 18S rRNA genes. Moreover, the ease with which specific sgRNA can be designed allows CCSAS to be used to explore the eukaryotic microbiome of almost any host organism. Thus, CCSAS can facilitate significant advances for investigations of the eukaryotic microbiome across a wide diversity of hosts.

## Methods

### Organisms and samples

Ten model organisms, human (*Homo sapiens*), salmon *(Salmo salar*), shrimp (*Solenocera crassicornis*), chicken (*Gallus gallus domesticus*), cow (*Bos taurus*), mouse (*Mus musculus*), fruit fly (*Drosophila melanogaster*), rock cress (*Arabidopsis thaliana*), oyster (*Crassostrea gigas*), and nematode (*Caenorhabditis elegans*), as well as nine species of protists and fungi were obtained from either commercial markets or laboratories at The University of British Columbia (Table S1). As well, eight samples of seven- to 28-day-old oyster spat, with sizes ranging between 0.4 and 1.0 mm, were obtained from a hatchery that was experiencing mortality events. The oyster spat were immediately frozen using liquid nitrogen following collection, and stored at −80 °C until analysis.

### Genomic DNA extraction

DNA from the model organisms, protists, fungi, and oyster spat were extracted using the DNeasy Blood & Tissue Kit (Qiagen) following the manufacturer’s directions, and quantified using the Qubit™ DNA HS Assay Kit (Invitrogen).

An artificial community of microeukaryotes was made by pooling equal amounts (~50 ng) of genomic DNA from each protist and fungus (Table S1).

### Design and synthesis of taxon-specific sgRNA

The specificity of CRISPR-Cas9 is determined by a 20-nt guide sequence within the sgRNA, which directs Cas9 to cut a target DNA at the 20-nt target site that is complementary to this guide sequence. Thus, the design of a taxon-specific sgRNA requires identifying a 20-nt gRNA-target-site oligonucleotide sequence in the host 18S rRNA gene, which is, absent in microeukaryotes. This taxon-specific 20-nt gRNA-target-site oligonucleotide sequence, reverse-complement to the sgRNA’s guide sequence, determines the specificity of the sgRNA and thereby the CRISPR-Cas action that is to cut 18S rRNA gene from the host but not from microeukaryotes. This taxon-specific 20-nt gRNA-target-site oligonucleotide sequence is used to synthesize the taxon-specific sgRNA using an EnGen™ sgRNA Synthesis Kit from New England Biolabs (NEB).

#### Obtaining the host 18S rRNA gene sequences

Prior to the design of the sgRNA, we obtained the 18S rRNA gene sequences of the host organisms for identifying gRNA target sites, and employed the following cloning and sequencing approaches:

For each host, 18S rRNA gene fragments were PCR amplified using the “universal” primers TAReuk454FWD1 and TAReukREV3 [54] to produce 380-450 bp amplicons that were sequenced to facilitate the design of gRNA-target-site oligos targeting each host. We selected these primers as they are among the most widely used to examine the diversity of microeukaryotic communities [99, 100]. Briefly, PCR was conducted in four separate reactions run at annealing temperatures of 45, 47, 48, or 49 °C, to ensure amplification of a 380-450 bp fragment from the V4 region of the 18S rRNA gene. Each 25 μL reaction mix was made with 1X PCR buffer (NEB), 4 mM MgCl_2_, 20 μg of Bovine Serum Albumin (NEB), 200 nM of each dNTP (Invitrogen), 0.4 μM of each primer, 0.5 U of Q5® high fidelity polymerase (NEB) and ~10 ng of genomic DNA template. As previously described [54], the initial denaturation and activation was at 95 °C for 5 min, followed by 10 cycles consisting of 95 °C for 30 s, 57 °C for 45 s, and 72 °C for 1 min, followed by 25 cycles of denaturation at 95 °C for 30 s, annealing at 45, 47, 48, or 49 °C for 45 s, elongation at 72 °C for 60 s, and a final elongation for 10 min at 72 °C. The PCR products from the four reactions were then pooled, and the 18S amplicons purified using Agencourt SPRI magnetic beads (Beckman Coulter) at a 1:1 (vol:vol) ratio of beads:DNA to remove fragments < 200 bp.

These purified amplicons were then cloned into pCR2-TOPO vectors (Invitrogen) using the TOPO TA Cloning Kit (Invitrogen). Four 18S rRNA gene clones from each model organism were sent for Sanger sequencing at the NAPS Unit sequencing facility at The University of British Columbia. These DNA sequences were then used to design the taxon-specific 20-nt gRNA-target-site sequences, which were used to synthesize the taxon-specific sgRNAs that guide Cas9 to cleave the host 18S sequences, as outlined below.

#### Design of the taxon-specific gRNA-target-site oligonucleotide sequences

We developed the R package CasOligo (https://github.com/kevinzhongxu/CasOligo) to design taxon-specific 20-nt gRNA-target-site oligonucleotide sequences, which allows sgRNA to recognize 18S sequences from specific taxa. Taxon-specific gRNA-target-site oligonucleotide sequences were designed for each model organism using the Cas9.gRNA.oligo1() function in CasOligo by providing a fasta file of the V4 region of the 18S rRNA gene from each organism that is amplified by the “universal” 18S primers, TAReuk454FWD1 and TAReukREV3 [54]. The same approach can be used to design taxon-specific gRNA-target-site oligonucleotide sequences for any host organism. First, Cas9.gRNA.oligo1() searches the forward and reverse strands of the 18S rRNA gene for 20-nt gRNA-target-site oligonucleotide sequences that are compatible with Cas9 nuclease; compatibility requires that the protospacer-adjacent-motif (PAM), NGG, is immediately adjacent to the 3′ downstream region of the 20-nt target-site sequence. Each of these 20-nt gRNA-target-site sequences is potentially a target for the combined actions of sgRNA and Cas9. Next, each potential gRNA-target-site sequence is searched against the SILVA SSU database for the V4 region of 18S rRNA genes, in order to determine if the sequence is absent in protistan and fungal microeukaryotes. If so, this gRNA-target-site sequence can be used to synthesize a sgRNA that will guide Cas9 to specifically cut the host 18S rRNA gene. The gRNA-target-site oligonucleotide sequences designed in this study are shown in Table S2.

#### Synthesis of sgRNA-template oligonucleotides

Once suitable taxon-specific 20-nt gRNA-target-site oligonucleotide sequences were identified, the sgRNA-template oligonucleotide sequences were obtained using the EnGen™ sgRNA Template Oligo Designer (https://nebiocalculator.neb.com/#!/sgrna), which adds a T7 promoter sequence at the 5′ end, and a 14-nt overlap sequence at the 3′ end of the 20-nt gRNA-target-site sequence. For our studies, this sgRNA-template oligonucleotide was synthesized by Integrated DNA Technologies (IDT), and diluted to 1 μM with molecular grade ultrapure water (Invitrogen).

#### Synthesis of sgRNA

The 1 μM sgRNA-template oligonucleotide was used as a DNA template to synthesize the sgRNA using the EnGen™ sgRNA synthesis kit, *S. pyogenes* (NEB) by following the manufacturer’s instructions. The resulting sgRNA was treated with amplification grade DNase I (Invitrogen) at room temperature for 15 min to remove any remaining DNA and then purified using a RNA Clean & Concentrator-25 Kit (Zymo Research) by following the manufacturer’s instructions. Finally, the fragment size of the sgRNA was assessed using an Agilent RNA 6000 Pico Kit (Agilent) and its concentration measured using a Qubit™ RNA HS Assay Kit (Invitrogen).

### Validation of the design of taxon-specific sgRNA

To validate the design of gRNA for taxon-specific cleavage, we first generated 18S amplicons for each model organism and the mock community of protists and fungi. Then, these 18S amplicons were used to ascertain the effect of CRISPR-Cas9, in conjunction with taxon-specific sgRNA, on cleavage of the amplicons. The results were visualized on a gel using a Bioanalyzer (Agilent) and assessed using quantitative PCR (qPCR) as detailed below.

#### Preparation of the host 18S amplicons

For each of the ten host organisms and the mock community of protists and fungi, 18S rRNA gene fragments were obtained using PCR with the “universal” primers TAReuk454FWD1 and TAReukREV3 [54] following the conditions detailed above. The 18S amplicons were purified using Agencourt SPRI magnetic beads (Beckman Coulter) at a 1:1 (vol:vol) ratio of beads:DNA.

#### DNA cleavage using CRISPR-Cas9

For each of the ten host organisms and the mock community of protists and fungi, the purified 18S amplicons were cut using Cas9 Nuclease, *S. pyogenes* (NEB) in the presence of a sgRNA, following the manufacturer’s directions. Briefly, the 10 μL reaction contained approximately 0.1 pmol of dsDNA, 1 pmol of sgRNA, and 1 pmol of Cas9, as well as 1x Cas9 reaction buffer to keep the molar ratio of Cas9:sgRNA:template DNA at 10:10:1. The reaction was incubated at 37 °C for 4 h in a thermocycler, followed by 70 °C for 10 min to deactivate the CRISPR-Cas9. For each sample, in parallel with the CRISPR-Cas9 treatment, we also prepared the reaction without CRISPR-Cas9 treatment, in which Cas9 nuclease and sgRNA were replaced with molecular grade ultrapure water (Invitrogen). Thus, each reaction of both treatments contained the same amount of template dsDNA (18S amplicons at 0.1 pmol) and was subjected to the same incubation conditions.

#### Visualization using gel electrophoresis

The size of the 18S rRNA gene fragments with and without CRISPR-Cas9 treatment was visualized by gel electrophoresis using a Bioanalyser (Agilent). Prior to loading into the gel, the Cas9-cut products (5 μL out of 10 μL) were treated with 1 mg/mL (final) Proteinase K (Invitrogen) at room temperature for 15 min to digest the Cas9 nuclease. Then, 1 to 2 μL of this proteinase-K-treated product was added into a well of an Agilent High Sensitive DNA Chip in a Bioanalyzer (Agilent) to visualize and verify cutting by CRISPR-Cas9.

#### Quantitative PCR

To determine the efficiency of CRISPR-Cas9 for eliminating host-derived 18S sequences, we used quantitative PCR (qPCR) and the primers TAReuk454FWD1 and TAReukREV3 (Table S3) that targets a 380-450 bp fragment of the V4 region of the 18S rRNA gene, to assess the proportion of 18S amplicons cut by Cas9. The 10 μL qPCR reactions contained 1 X SsoFast™ EvaGreen® Supermix (Bio-Rad), 0.5 μM of each primer, and a 1 μL 1/10000 dilution of DNA template consisting of amplified products, either with or without the addition of Cas9. Thermal cycling was done in a CFX96 real-time PCR detection system (Bio-Rad) with the following program: 3 min denaturation at 95 °C, followed by 40 cycles of denaturation at 95 °C for 30 s, and annealing and extension at 49 °C for 30 s. Nine, tenfold serially diluted standards (ranging from 5 × 10^0^ to 5 × 10^9^ molecules per mL) were run in duplicate along with two no-template control reactions containing 1 μL of nuclease-free water. The amplicon standards were made from a cloned 18S rRNA gene fragment amplified from a culture of the prasinophyte microalga, *Micromonas pusilla*, using the primer set TAReuk454FWD1/TAReukREV3 [54]. The amplicons were purified using a MiniElute® PCR Purification Kit (Qiagen), and quantified using a Qubit® dsDNA High Sensitivity Assay Kit (Invitrogen). The size of the amplicon was checked using gel-electrophoresis, and the qPCR melting curves were used to confirm that the fluorescence signal corresponded to a single-sized DNA fragment. The qPCR amplification efficiency was between 0.95 and 1.05 for the cloned amplicons (with *r* > 0.98, *n* = 9).

### Sequencing library preparation using CRISPR-Cas Selective Amplicon Sequencing (CCSAS)

To profile host-associated eukaryotic microbiomes, we developed CRISPR-Cas Selective Amplicon Sequencing (CCSAS), which combines the use of CRISPR-Cas9 and universal 18S primers to prepare a sequencing library that is compatible with Illumina sequencing platforms. The method uses a taxon-specific sgRNA to guide Cas9 nuclease to selectively cleave 18S rRNA gene sequences from metazoa and plants, which then can be removed by size selection with SPRI beads; sequences from microeukaryotes are left intact, and can be amplified by PCR. Therefore, CCSAS allows high-resolution profiling of host-associated eukaryotic microbiomes with relatively low sequencing effort. In this study, we present CCSAS (Fig. 2); the two-step CRISPR-Cas procedure first uses Cas9 to cut the host gene encoding 18S rRNA gene, followed by a second cut of any host-derived 18S amplicons. Details of the method are provided below.

#### Cas9 cutting of host genomic DNA

Genomic DNA of the host was cut using Cas9 Nuclease, *S. pyogenes* (NEB) following the manufacturer’s directions. Briefly, a 10-μL reaction mix containing approximately 0.1 pmol of genomic DNA, 1 pmol of sgRNA and 1 pmol of Cas9, as well as 1x Cas9 reaction buffer to keep the molar ratio of Cas9:sgRNA:template DNA at 10:10:1 was incubated at 37 °C for 4 h in a thermocycler.

#### The first PCR and size selection

The Cas9-cleaved genomic DNA was used as a template in the first PCR to generate 380-450 bp amplicons from the V4 region of the 18S rRNA gene that are depleted in host sequences. To ensure representative amplification of 18S sequences from microeukaryotes, four parallel PCR reactions were run at different annealing temperatures (45, 47, 48, or 49 °C), using the “universal” 18S primers TAReuk454FWD1-Nxt and TAReukREV3-Nxt (Table S3). Compared to TAReuk454FWD1 and TAReukREV3 [54], this modified primer set contained overhang adapter sequences (Table S3), which are compatible with Illumina indexes and sequencing adapters. These adapters allowed for a second PCR to append Illumina Nextera XT indexes to each side of the amplicons as forward and reverse primers, thus creating a dual-indexed library. This dual-indexed library preparation approach is adapted from Illumina [101].

Details on the first PCR reactions are as follows. Briefly, each 25 μL reaction mix contained 1X PCR buffer (NEB), 4 mM MgCl_2_, 20 μg of Bovine Serum Albumin (NEB), 200 nM of each dNTP (Invitrogen), 0.4 μM of each primer, 0.5 U of Q5® high fidelity polymerase (NEB), and 5 μL of the Cas9-cleaved genomic DNA. Because the reverse primer is 2 bp shorter than the forward primer and has a lower annealing temperature, we used the two-step PCR approach of Stoeck et al. [54], in which there is an initial ten PCR cycles at an annealing temperature where only the forward primer will bind and amplify, followed by 25 cycles at one of four lower annealing temperatures (45, 47, 48, or 49 °C) where both forward and reverse primers amplify. The program has an initial denaturation and activation at 95 °C for 5 min, followed by ten, three-step cycles consisting of 94 °C for 30 s, 57 °C for 45 s, and 72 °C for 1 min, followed by 25 cycles of denaturation at 94 °C for 30 s, annealing at either 45, 47, 48, or 49 °C for 45 s and elongation at 72 °C for 60 s, with a final elongation for 10 min at 72 °C. At the end, the PCR product of the four reactions was pooled together. Then, amplicons were size-selected and purified using magnetic Agencourt SPRI beads (Beckman Coulter) at an 0.8:1 (vol:vol) ratio of beads:DNA to remove fragment < 300bp.

#### Cas9 cutting of the 18S amplicons

To further remove 18S host amplicons, the size-selected amplicons described above were cut again using Cas9 Nuclease, *S. pyogenes* (NEB). Briefly, the 10 μL reaction contained approximately 0.1 pmol of DNA amplicons, 1 pmol of sgRNA, 1 pmol of Cas9, 1x Cas9 reaction buffer to keep the molar ratio of Cas9:sgRNA:template DNA at 10:10:1. The reaction was incubated at 37 °C for 4 h in a thermocycler.

#### The 2nd PCR and size selection

The product of the second Cas9-cut was used as the template for a second PCR (index PCR) to generate the indexed amplicons libraries. The 50-μL reaction mix of the second PCR comprised 1X PCR buffer (NEB), 4 mM MgCl_2_, 200 nM of each dNTP (Invitrogen), 5 μL of each index primer (N7XX and S5XX of Nextera® XT Index Kit, Illumina), 1 U of Q5® high fidelity polymerase (NEB), and 5 μL of the product of the second Cas9 cut. The second PCR (index PCR) consisted of an initial denaturation and activation at 95 °C for 3 min, followed by 29 three-step cycles consisting of 95 °C for 30 s, 55 °C for 30 s, and 72 °C for 30 s, and a final elongation for 10 min at 72 °C. The indexed amplicons generated by the second PCR were size-selected and purified using magnetic Agencourt SPRI beads (Beckman Coulter) at a ratio of 0.8:1 (vol:vol) for beads:DNA to remove fragments < 300 bp.

During size selection with SPRI magnetic beads, the bead:DNA ratio depends on the size of the fragments that need to be separated. As the size of the fragments generated by cutting the ~424-bp metazoan 18S rRNA gene sequences will vary depending on the cut site, the beads:DNA ratio of a specific sgRNA may need to be optimized to remove all of the cleaved fragments. It is important to remove sequence fragments generated by amplification of the cleaved host 18S rRNA genes, as these can reduce sequencing efficiency.

### Sequencing library preparation for amplicons generated using universal 18S primers

Sequencing libraries for 18S amplicons generated using the “universal” 18S primers, and not cut using CRISPR-Cas9, were prepared using protocols adapted from Illumina [101]. Briefly, two successive runs of PCR were performed as follows: For the first PCR, 29 cycles of amplification using the modified primers TAReuk454FWD1-Nxt and TAReukREV3-Nxt (Table S3) were used to generate 380 to 450 bp amplicons of the V4 region of the 18S rRNA genes. The reaction conditions for the first PCR were as detailed above for the first CCSAS PCR, except that there was about 5 ng of genomic DNA in the sample. The amplicons were purified using magnetic Agencourt SPRI beads (Beckman Coulter) at a ratio of 1:1 (vol:vol) for beads:DNA to remove fragments < 200 bp.

Five microliters of the purified amplicons from the first PCR were used as templates for the second PCR (index PCR). The PCR reactions and conditions for the index PCR were the same as above the second CCSAS PCR, except here the PCR amplification cycle was reduced to be 16 cycles. The amplicon libraries generated were purified using magnetic Agencourt SPRI beads (Beckman Coulter) at a ratio of 1:1 (vol:vol) for beads:DNA to remove fragments < 200 bp.

### Sequencing library preparation for amplicons generated using the blocking primers

Preparation of the sequencing library for the 18S amplicons obtained using blocking primers was similar to that described above, except that the first PCR used the primer set 18SV4-F-Nxt/18SV4-R-Nxt and the oyster-blocking primer 18SV4-Block-oyster (Table S3), which was adapted from Clerissi et al. [57], to amplify a ~377 bp fragment of 18S rRNA gene that is specific to microeukaryotes but not Pacific oysters. This 30-nt oyster-blocking primer was modified at the 3′ end with theSpacer C3 CPG (3 hydrocarbons) and contained a 10-bp overlap with the reverse primer 18SV4-R-Nxt, which prevents the amplification of the 18S rRNA gene from Pacific oysters, and thus enriches the proportion of amplicons from microeukaryotes [57]. In the first PCR, the 25-μL reaction mix comprised 1X PCR buffer (NEB), 4 mM MgCl_2_, 20 μg of Bovine Serum Albumin (NEB), 200 nM of each dNTP (Invitrogen), 0.4 μM of primer 18SV4-F-Nxt, 0.4 μM of primer 18SV4-R-Nxt, 1.2 μM of the oyster-blocking primer 18SV4-Block-oyster, 0.5 U of Q5® high-fidelity polymerase (NEB), and approximately 5 ng of genomic DNA. The PCR cycling was as per Clerissi et al. [57], which included an initial incubation of 15 min at 96 °C followed by 35 cycles of denaturation at 96 °C for 30 s, annealing at 52 °C for 30 s and elongation at 72 °C for 60 s, and a final elongation for 10 min at 72 °C. The first PCR product was purified using magnetic Agencourt SPRI beads (Beckman Coulter) at a ratio of 1:1 (vol:vol) for beads:DNA to remove fragments < 200 bp (e.g., dimers). The amplicon libraries were completed as described above, with a 16-cycle index PCR to add a Nextera® XT index (Illumina) to each 3′ and 5′ end of the amplicons.

### Sequencing library preparation for amplicons generated using non-metazoan primers

The non-metazoan primers, UNonMet primers [58], and a two-step nested-PCR were used to generate 18S amplicons from non-metazoan eukaryotes following del Campo et al. [70]. The first step of the nested-PCR uses the primers 18s-EUK581-F and 18s-EUK1134-R [58] (Table S3) to generate ~600-bp 18S rRNA gene fragments from microeukaryotes. Then, these fragments are used in a second PCR with the universal V4 primer set E572F-Nxt/E1009R-Nxt [102] (Table S3) to amplify a ~440-bp 18S rRNA gene fragment to which overhanging adapter sequences (Table S3) are added that are compatible with the Illumina indexes and sequencing adapters. Finally, a third PCR is used to add a Nextera® XT index (Illumina) to each 3′ and 5′ end of the amplicons.

In the first PCR, the 25 μL reaction mix comprised 1X PCR buffer (NEB), 4 mM MgCl_2_, 20 μg of Bovine Serum Albumin (NEB), 200 nM of each dNTP (Invitrogen), 0.4 μM of each primer, 0.5 U of Q5® high-fidelity polymerase (NEB), and approximately 5 ng of genomic DNA. The initial denaturation of 2 min at 98 °C was followed by 25 cycles of denaturation at 98 °C for 30 s, annealing at 51.5 °C for 30 s and elongation at 72 °C for 60 s, and a final elongation for 10 min at 72 °C. The first PCR product was purified using magnetic Agencourt SPRI beads (Beckman Coulter) at a ratio of 1:1 (vol:vol) for beads:DNA to remove fragments < 200bp (e.g., dimers).

In the second PCR, adapted from Comeau et al. [102], the 25 μL reaction mix comprised 1X PCR buffer (NEB), 4 mM MgCl_2_, 20 μg of Bovine Serum Albumin (NEB), 200 nM of each dNTP (Invitrogen), 0.4 μM of each primer, 0.5 U of Q5® high-fidelity polymerase (NEB), and approximately 5 ng of the purified 1st PCR amplicons. There initial denaturation of 2 min at 98 °C was followed by 20 cycles of denaturation at 98 °C for 10 s, annealing at 55 °C for 30 s and elongation at 72 °C for 30 s, and a final elongation for 10 min at 72 °C. The PCR product was purified using magnetic Agencourt SPRI beads (Beckman Coulter) at a ratio of 1:1 (vol:vol) for beads:DNA to remove fragments < 200 bp (e.g., dimers).

Last, the amplicon libraries were completed as described above, with a 16-cycles of PCR to add the Nextera® XT index (Illumina) to the 3′ and 5′ ends of the amplicons.

### Next-generation sequencing and data analysis

The DNA concentrations of the 18S amplicon sequencing libraries that were prepared using “universal” 18S primers, non-metazoan primers, blocking primers, or the CCSAS method were measured using the Qubit® dsDNA High Sensibility Assay Kit (Invitrogen). The fragment size for each type of library was determined using an Agilent bioanalyzer with the High Sensitive DNA Chip (Agilent). Equimolar amounts of these barcoded and purified amplicon sequencing libraries were pooled and sequenced at the BRC Sequencing Core at The University of British Columbia using MiSeq Illumina 2 × 300bp chemistry.

Sequences were processed and analyzed using QIIME version 1.9 [103]. Briefly, sequences were de-multiplexed by their forward and reverse indexes, and the paired-end reads merged using PEAR version 1.10.4 [104]. Then, sequences from different samples were pooled, and Uclust [105] was used for OTU picking with 99% nucleotide sequence similarity. Taxonomy was assigned for representative OTU sequences using the Uclust consensus taxonomy assigner and the SILVA SSU database [80] (*version v132, released on 13 December 2017*) at a 90% confidence cutoff. The samples were normalized by analyzing the relative abundance for each OTU or taxon as the proportion of all sequences within a sample. The downstream analysis was conducted in R v3.5.3 [106] using packages such as Phyloseq version 1.26.1 [107] and the figures were generated using ggplot2 version 3.3.0 [108] and metacoder [109].

To compare microeukaryotic community structures between samples and methods, the principal coordinate analyses (PCoA) were performed on the ordination of the weighted Unifrac metrics [110] that were based on the presence/absence and relative abundance of OTUs. The dissimilarity of the microeukaryotic community composition between methods was examined using PERMANOVA analysis [111] with the adonis function and Bray-Curtis method in R package Vegan v.2.5 [112] and Microbiome version 1.13.12 [113]. The similarity of the microeukaryotic community composition between methods was examined using ANOSIM analysis [114] with the anosim function and Jaccard method in R package Vegan v.2.5 [112].

Linear discriminant analysis effect size (LEfSe) [115] was conducted to identify oyster associated micro-eukaryotic taxa that were differentially abundant between three methods, and was calculated with default setting using the Galaxy modules provided by the Huttenhower lab. Briefly, LEfSe used the two-tailed nonparametric Kruskal-Wallis test to evaluate the significance of differences in taxonomic abundance between three methods. Then, a set of pairwise tests among three methods was performed using the unpaired Wilcoxon test. In the end, the linear discriminant analysis (LDA) was conducted to estimate the effect size of each differentially abundant taxa [115].

## Supplementary Information

**Additional file 1: Table S1.** List of organisms used in this study.

**Additional file 2: Table S2.** List of the 20-nt sgRNA-target-site oligonucleotide sequences designed for cutting V4 region of 18S rRNA genes of ten model organisms using CRISPR-Cas9.

**Additional file 3: Table S3.** List of primers used in this study.

**Additional file 4: Figure S1.** Percentage of intact 18S amplicons remaining from the model organisms after cutting with one-step CRISPR-Cas9. The concentration of intact 18S amplicons was measured using Quantitative PCR for samples both with and without CRISPR-Cas9 treatment. The portion of remaining intact 18S amplicons was determined by dividing the concentration 18S amplicons in sample with CRISPR-Cas9 treatment by that of without CRISPR-Cas9 treatment. The labels on the X-axes indicate the ID of the taxon-specific sgRNA and its corresponding host.

**Additional file 5: Figure S2.** Eukaryotic taxa representing >1% of the sequences revealed by deep-sequencing of the 18S rRNA amplicons for oyster spat samples, using "universal" 18S primers (Table S3), non-metazoan primers (Table S3), blocking primers (Table S3), or CRISPR-Cas Selective Amplicon Sequencing (**CCSAS**) combining "universal" 18S primers and CRISPR-Cas9 with pacific-oyster-specific sgRNA m258 (Table S2).

**Additional file 6: Figure S3.** Distribution of the number of gRNA-target-sites of each metazoan and plant species from the SILVA SSU database v119 [80]. These gRNA-target-site oligonucleotide sequences were identified, using the Cas9.gRNA.oligo1() algorithm, from the V4 region of the 18S rRNA gene that is flanked by the 18S "universal" primer set TAReuk454FWD1 / TAReukREV3 [54], and are used for designing and synthesizing the CRISPR-Cas9-compatible sgRNA. The taxon-specific gRNA-target-sites allows the design of the sgRNA to taxon-specifically cut the 18S rRNA gene sequence of a metazoan or plant host but not microeukaryotes (protists and fungi) using CRISPR-Cas9.

**Additional file 7: Figure S4.** Summary of the number of eukaryotic species at each D7 taxonomic level that the sgRNA can cut at the V4 region of the 18S rRNA genes that are flanked by the 18S "universal" primer set TAReuk454FWD1 / TAReukREV3 [54]. These nine sgRNAs, which are among 205242 unique taxon-specific sgRNA designed from the SILVA SSU database (*version 119*) [80] using CasOligo, are selected to show that some sgRNAs can target more than 1000 species and broad taxonomic groups based on an *in-silico* analysis (*i.e.* 100% match to the 18S rRNA gene sequences of the metazoan host at the gRNA-target-site, but no match for protists and fungi). Taxon names on the left side of the panel are shown as SILVA taxonomic hierarchy with levels ranging from D0 (kingdom) to D7. The D7 taxonomic level comprises eukaryotic classes and families.

**Additional file 8: Figure S5.** Relative abundances of ciliates from the subclass Scuticociliatia in eight oyster samples revealed using deep-sequencing of 18S amplicons of rRNA genes using non-metazoan primers (NM), blocking primers (BP) and CRISPR-Cas Selective Amplicon Sequencing (CCSAS). The relative abundances of scuticociliates are presented as a barplot (**a**), and as a heatmap showing the genus-level relative abundances of scuticociliates (**b**).

## Data Availability

All next-generation sequencing data generated in this study have been deposited in the NCBI Sequence Read Archive (SRA) under the accession numbers SRR13658714 to SRR13658745. The Sanger cloning-sequencing data were deposited in GenBank under the accession numbers MT328571 to MT328580 for 18S rRNA gene sequences of ten model organisms. All related scripts, functions, and algorithms for designing gRNA-target-site oligonucleotide sequences are included in the custom R package: CasOligo (https://github.com/kevinzhongxu/CasOligo). The gRNA-target-sites database was included in the CasOligo package, as well. The authors declare that all other data supporting the findings of this study are available within the paper and/or the associated supplementary files.

## Abbreviations

CRISPR: Clustered regularly interspaced short palindromic repeats
Cas: CRISPR-associated protein
CCSAS: CRISPR-Cas Selective Amplicon Sequencing
gRNA: Guide RNA
sgRNA: Single-guide RNA
dsDNA: Double-stranded DNA
ssDNA: Single-stranded DNA
nt: Nucleotide
crRNA: Crispr RNA
tracrRNA: Trans-activating RNA
V4 region of the 18S rRNA gene: Variable region 4 of the 18S rRNA gene
PCR: Polymerase chain reaction
OTU: Operational taxonomic unit
SSU: Small subunit
dNTP: Deoxyribonucleotide triphosphate
bp: Base pairs
