# Supplementary material for: Revealing the composition of the eukaryotic microbiome of oyster spat by CRISPR-Cas Selective Amplicon Sequencing (CCSAS)
Source: Microbiome. 2021 Nov 26;9:230. doi: 10.1186/s40168-021-01180-0 (PMC8620255; doi:10.1186/s40168-021-01180-0)
Supplement: Supplementary file 2 — Additional file 1: Table S1. List of organisms used in this study. [file 40168_2021_1180_MOESM1_ESM.docx]

**Table S1** List of organisms used in this study.
