# Supplementary material for: Revealing the composition of the eukaryotic microbiome of oyster spat by CRISPR-Cas Selective Amplicon Sequencing (CCSAS)
Source: Microbiome. 2021 Nov 26;9:230. doi: 10.1186/s40168-021-01180-0 (PMC8620255; doi:10.1186/s40168-021-01180-0)
Supplement: Supplementary file 3 — Additional file 2: Table S2. List of the 20-nt sgRNA-target-site oligonucleotide sequences designed for cutting V4 region of 18S rRNA genes of ten model organisms using CRISPR-Cas9. [file 40168_2021_1180_MOESM2_ESM.docx]

**Table S2** List of the 20-nt sgRNA-target-site oligonucleotide sequences designed for cutting V4 region of 18S rRNA genes of ten model organisms using CRISPR-Cas9.
