# Supplementary material for: Revealing the composition of the eukaryotic microbiome of oyster spat by CRISPR-Cas Selective Amplicon Sequencing (CCSAS)
Source: Microbiome. 2021 Nov 26;9:230. doi: 10.1186/s40168-021-01180-0 (PMC8620255; doi:10.1186/s40168-021-01180-0)
Supplement: Supplementary file 4 — Additional file 3: Table S3. List of primers used in this study. [file 40168_2021_1180_MOESM3_ESM.docx]

**Table S3** List of primers used in this study.
