# Supplementary material for: Revealing the composition of the eukaryotic microbiome of oyster spat by CRISPR-Cas Selective Amplicon Sequencing (CCSAS)
Source: Microbiome. 2021 Nov 26;9:230. doi: 10.1186/s40168-021-01180-0 (PMC8620255; doi:10.1186/s40168-021-01180-0)
Supplement: Supplementary file 5 — Additional file 4: Figure S1. Percentage of intact 18S amplicons remaining from the model organisms after cutting with one-step CRISPR-Cas9. The concentration of intact 18S amplicons was measured using Quantitative PCR for samples both with and without CRISPR-Cas9 treatment. The portion of remaining intact 18S amplicons was determined by dividing the concentration 18S amplicons in sample with CRISPR-Cas9 treatment by that of without CRISPR-Cas9 treatment. The labels on the X-axes indicate the ID of the taxon-specific sgRNA and its corresponding host. [file 40168_2021_1180_MOESM4_ESM.docx]

**Fig. S1** Percentage of intact 18S amplicons remaining from the model organisms after cutting with one-step CRISPR-Cas9. The concentration of intact 18S amplicons was measured using Quantitative PCR for samples both with and without CRISPR-Cas9 treatment. The portion of remaining intact 18S amplicons was determined by dividing the concentration 18S amplicons in sample with CRISPR-Cas9 treatment by that of without CRISPR-Cas9 treatment. The labels on the X-axes indicate the ID of the taxon-specific sgRNA and its corresponding host.
