# Supplementary material for: Revealing the composition of the eukaryotic microbiome of oyster spat by CRISPR-Cas Selective Amplicon Sequencing (CCSAS)
Source: Microbiome. 2021 Nov 26;9:230. doi: 10.1186/s40168-021-01180-0 (PMC8620255; doi:10.1186/s40168-021-01180-0)
Supplement: Supplementary file 6 — Additional file 5: Figure S2. Eukaryotic taxa representing >1% of the sequences revealed by deep-sequencing of the 18S rRNA amplicons for oyster spat samples, using "universal" 18S primers (Table S3), non-metazoan primers (Table S3), blocking primers (Table S3), or CRISPR-Cas Selective Amplicon Sequencing (CCSAS) combining "universal" 18S primers and CRISPR-Cas9 with pacific-oyster-specific sgRNA m258 (Table S2). [file 40168_2021_1180_MOESM5_ESM.docx]

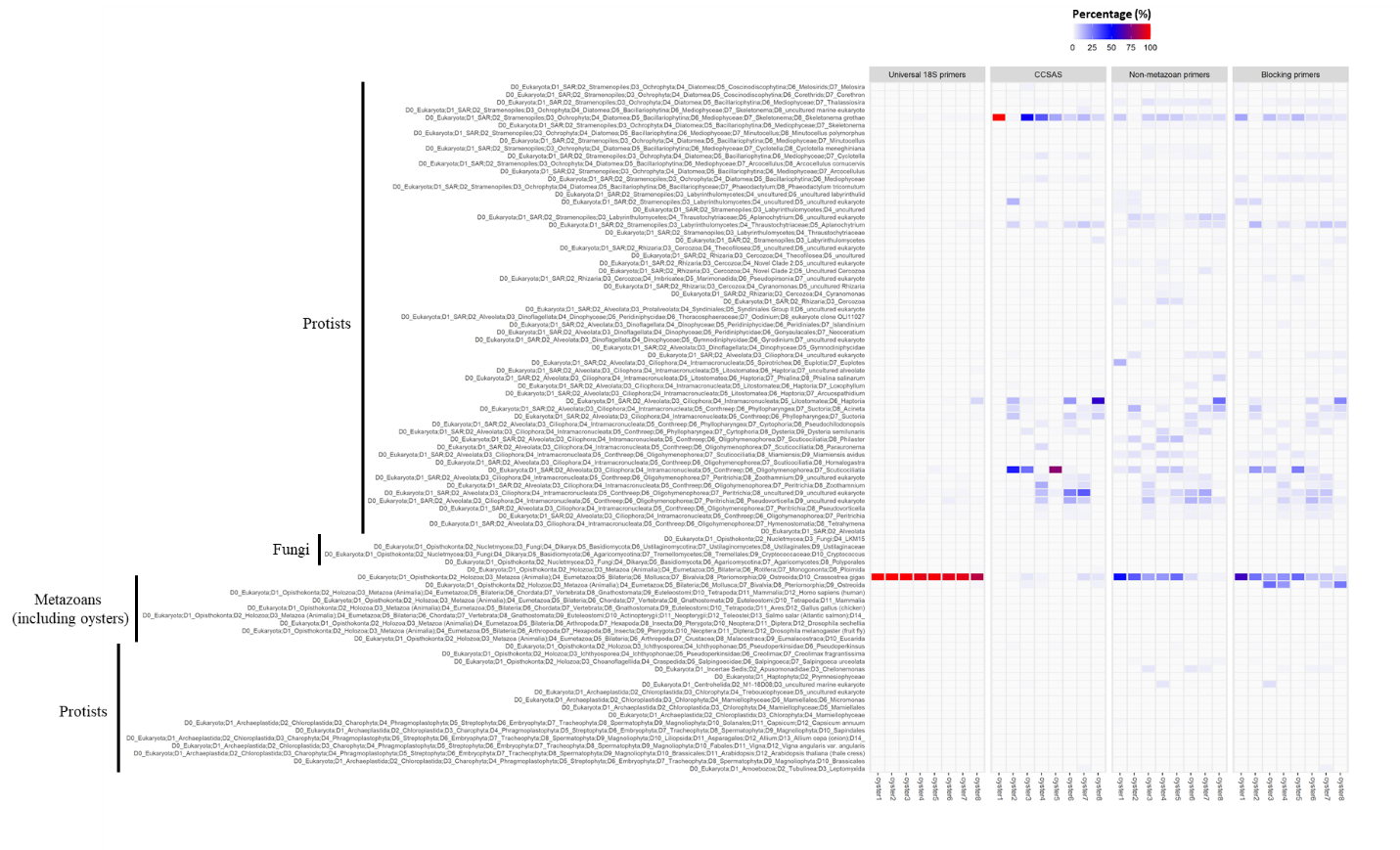


**Fig. S2** Eukaryotic taxa representing >1% of the sequences revealed by deep-sequencing of the 18S rRNA amplicons for oyster spat samples, using "**universal" 18S primers** (Table S3), **non-metazoan primers** (Table S3), **blocking primers** (Table S3), or CRISPR-Cas Selective Amplicon Sequencing (**CCSAS**) combining "universal" 18S primers and CRISPR-Cas9 with pacific-oyster-specific sgRNA m258 (Table S2).
