# Supplementary material for: Revealing the composition of the eukaryotic microbiome of oyster spat by CRISPR-Cas Selective Amplicon Sequencing (CCSAS)
Source: Microbiome. 2021 Nov 26;9:230. doi: 10.1186/s40168-021-01180-0 (PMC8620255; doi:10.1186/s40168-021-01180-0)
Supplement: Supplementary file 7 — Additional file 6: Figure S3. Distribution of the number of gRNA-target-sites of each metazoan and plant species from the SILVA SSU database v119 [80]. These gRNA-target-site oligonucleotide sequences were identified, using the Cas9.gRNA.oligo1() algorithm, from the V4 region of the 18S rRNA gene that is flanked by the 18S "universal" primer set TAReuk454FWD1 / TAReukREV3 [54], and are used for designing and synthesizing the CRISPR-Cas9-compatible sgRNA. The taxon-specific gRNA-target-sites allows the design of the sgRNA to taxon-specifically cut the 18S rRNA gene sequence of a metazoan or plant host but not microeukaryotes (protists and fungi) using CRISPR-Cas9. [file 40168_2021_1180_MOESM6_ESM.docx]

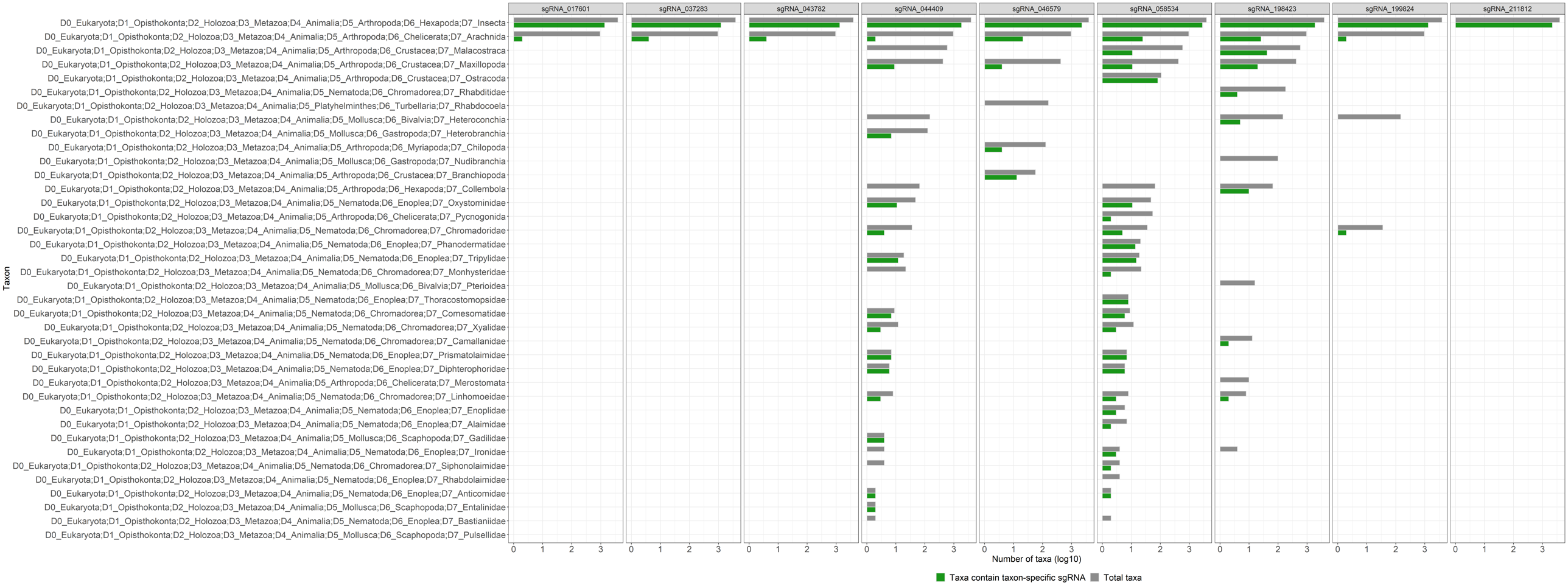


**Fig. S4** Summary of the number of eukaryotic species at each D7 taxonomic level that the sgRNA can cut at the V4 region of the 18S rRNA genes that are flanked by the 18S "universal" primer set TAReuk454FWD1 / TAReukREV3 [54]. These nine sgRNAs, which are among 205242 unique taxon-specific sgRNA designed from the SILVA SSU database (*version 119*) [81] using CasOligo, are selected to show that some sgRNAs can target more than 1000 species and broad taxonomic groups based on an *in-silico* analysis (*i.e.* 100% match to the 18S rRNA gene sequences of the metazoan host at the gRNA-target-site, but no match for protists and fungi). Taxon names on the left side of the panel are shown as SILVA taxonomic hierarchy with levels ranging from D0 (kingdom) to D7. The D7 taxonomic level comprises eukaryotic classes and families.
