# Supplementary material for: Revealing the composition of the eukaryotic microbiome of oyster spat by CRISPR-Cas Selective Amplicon Sequencing (CCSAS)
Source: Microbiome. 2021 Nov 26;9:230. doi: 10.1186/s40168-021-01180-0 (PMC8620255; doi:10.1186/s40168-021-01180-0)
Supplement: Supplementary file 9 — Additional file 8: Figure S5. Relative abundances of ciliates from the subclass Scuticociliatia in eight oyster samples revealed using deep-sequencing of 18S amplicons of rRNA genes using non-metazoan primers (NM), blocking primers (BP) and CRISPR-Cas Selective Amplicon Sequencing (CCSAS). The relative abundances of scuticociliates are presented as a barplot (a), and as a heatmap showing the genus-level relative abundances of scuticociliates (b). [file 40168_2021_1180_MOESM8_ESM.docx]

**Fig. S5** Relative abundances of ciliates from subclass Scuticociliatia in eight oyster samples revealed using deep-sequencing of 18S amplicons of rRNA genes using non-metazoan primers (NM), blocking primers (BP) and CRISPR-Cas Selective Amplicon Sequencing (CCSAS). The relative abundances of scuticociliates are presented as barplots (**a**), and as a heatmap showing the genus-level relative abundances of scuticociliates (**b**).
